# Surgical treatment for mediastinitis after endobronchial ultrasound-guided transbronchial needle aspiration: 2 case reports

**DOI:** 10.1016/j.ijscr.2020.11.068

**Published:** 2020-11-28

**Authors:** Sung Joon Han, Yooyoung Chong, Man-shik Shim, Woosik Han, Hyun Jin Cho, Shin Kwang Kang, Jae Hyeon Yu

**Affiliations:** Department of Thoracic and Cardiovascular Surgery, Chungnam National University Hospital, Chungnam National University School of Medicine, Munhwa-ro 282, Jung-gu, Daejeon, 35015, South Korea

**Keywords:** Mediastinitis, Endobronchial ultrasound guided transbrochial needle aspiration, Abscess, Case report

## Abstract

We report two cases of severe mediastinitis accompanied by abscess due to endobronchial ultrasound-guided transbronchial needle aspiration (EBUS-TBNA), that were successfully treated by effective surgical drainage. A 68-year-old woman was referred to our hospital due to chest discomfort and high fever after EBUS-TBNA, and a 54-year-old man was referred due to general weakness, chills, and high fever after the same procedure. Both were diagnosed with EBUS-related mediastinitis and discharged after surgical treatment. Similar to previous reports, the importance of surgical procedures for mediastinitis caused by EBUS-TBNA was suggested. Further research and establishment of guidelines on this matter is necessary.

## Highlights

• Mediastinitis by endobronchial ultrasound-guided transbronchial needle aspiration.
• EBUS-related mediastinitis is treated effectively by surgical drainage.
• Combination of surgery and medical treatment can be very effective in the treatment of inflammation caused by EBUS.

## 1 Introduction

With the recent development in radiology, the diagnosis rates of various thoracal diseases are continuously increasing, and the reporting of not only malignant diseases such as lung cancer and mediastinal tumors, but also diseases such as lymphadenopathy is increasing due to the wide introduction of screening programs that use low-dose computed tomography (CT).

Previously, when an enlargement of a mediastinal lymph node was discovered, biopsy using mediastinoscopy or video-assisted thoracic surgery (VATS) was performed; however, recently endobronchial ultrasound-guided transbronchial needle aspiration (EBUS-TBNA) has been relatively widely used [1]. This is a minimally invasive method that allows access to mediastinum and hilum, and has the advantage of not only staging lung cancer, but also allowing biopsy of the lesion with a relatively low risk of complications. Even though procedure-related complications exist [2], they are not significant compared with the safety and efficacy of the method [3]. We report two cases that were successfully treated by effective surgical drainage of severe mediastinitis accompanied by abscess due to the EBUS-TBNA.

## 2 Presentation of case

### 2.1 Case 1

A 68-year-old woman was referred to our hospital due to a growing shadow in the right paratracheal and hilar area on the chest X-ray performed during the medical examination. She was a non-smoker and had a history of tuberculosis (TB) 40 years ago. A CT scan revealed two lobulated masses with homogeneous contrast enhancement in the right paratracheal and hilar area; however, no lung lesions were observed. EBUS-TBNA of the lesion was performed under deep sedation by a well-trained bronchoscopist. The patient was discharged without complications 1 day after the procedure. The amount of specimen was sufficient to analyze histological results, and no evidence of malignancy was found.

Eight days after the EBUS-TBNA, the patient was admitted to our emergency room due to chest discomfort and high fever. The vital signs were: systolic blood pressure (BP) 100 mm Hg, diastolic BP 60 mm Hg, heart rate 82/min, respiratory rate 16/min, and peak fever 38.4 °C. The blood test results revealed a white blood cell count of 12110/μL and C-reactive protein of 25.3 mg/dL.

**Fig. 1:**
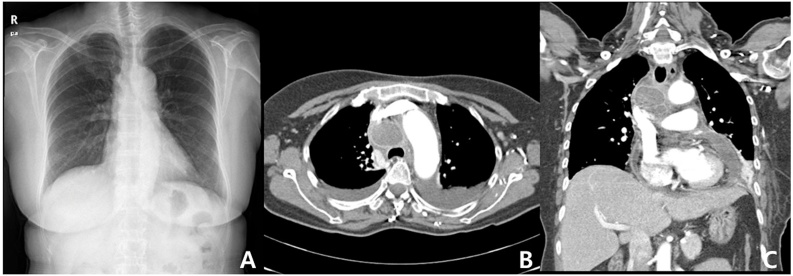
(A) Chest radiograph, performed 8 days after EBUS-TBNA, shows pleural effusion in both lungs, enlarged heart margin, and the size of the shadow previously observed in right hilar region increased further; (B, C) chest CT, performed 8 days after EBUS-TBNA, shows large amount of pericardial effusion, enlargement of the right lower paratracheal lymph nodes, and a multiloculated mediastinal abscess with gas formation along with the appearance of pleural effusion in both lungs.

On a chest X-ray, pleural effusion of both lung fields and enlarged heart margin were revealed, in addition to a previously observed shadow in the right hilar area that had increased in size (Fig. 1A). A portable cardiac echocardiogram was performed. Although the ejection fraction of the left ventricle was within the normal range, a large pericardial effusion was observed. Pig tail catheter was inserted through percutaneous pericardiocentesis and 400 mL of fluid was drained, and symptoms were slightly improved. On a chest CT, enlargement of the right lower paratracheal lymph nodes and a multiloculated mediastinal abscess with gas formation (Fig. 1B, C) were observed. The patient underwent surgery for debridement and drainage along with lymph node dissection on the same day.

The operation was initiated with VATS under general anesthesia; adhesion of the whole lung and severe adhesion of the target lesion to surrounding structures were revealed; therefore, we converted to thoracotomy. Purulent fluid was contained in the thoracic cavity when we approached the target lesion, and a thick layer was found around the swollen lymph node; therefore, dissection was performed using surgical electrocautery and energy devices. Upon detachment, a large amount of pus-like fluid was secreted, and cytologic examination and culture were performed. Conglomerated and necrotic lesions seen on the CT were removed as much as possible, and biopsy was performed to confirm the pathology. The surgery was completed after massive saline irrigation, a 28-French chest tube was inserted into the thoracic cavity, and a 28-French right-angled chest tube was inserted into the pericardial space for effective drainage of the pericardial effusion. After the operation, the patient had stable vital signs without fever and gradually recovered. The pathological examination of the biopsy obtained during the surgery revealed inflammation, without other abnormal findings. During the hospitalization period, additional CT performed to check the changes of lesion revealed reduction in size, and the blood test results were within the normal range. The patient was discharged without any complications.

### 2.2 Case 2

A 54-year-old man was referred to the otorhinolaryngology department in our hospital for examination due to voice change that started 6 months ago. The patient had a history of TB 30 years ago and was taking medication for arterial hypertension and diabetes. CT scan of both sides confirmed multiple lymphadenopathies with calcific foci in the mediastinum, and EBUS-TBNA was performed for biopsy. Target lesions were the right paratracheal and subcarinal lymph nodes. The patient was discharged the day after the procedure without complications, and histological examination revealed few lymphoid cells with necrotic debris.

The patient was admitted to the emergency room of our hospital 21 days after the EBUS-TBNA due to general weakness, chills, and high fever of 39.3 °C. Systolic BP was 107 mm Hg, diastolic BP was 67 mm Hg, heart rate was 82/min, and respiration rate was 18/min. The blood test results revealed a white blood cell count of 7720/μL, C-reactive protein of 26.4 mg/dL, and procalcitonin of 58.89 ng/dL.

**Fig. 2:**
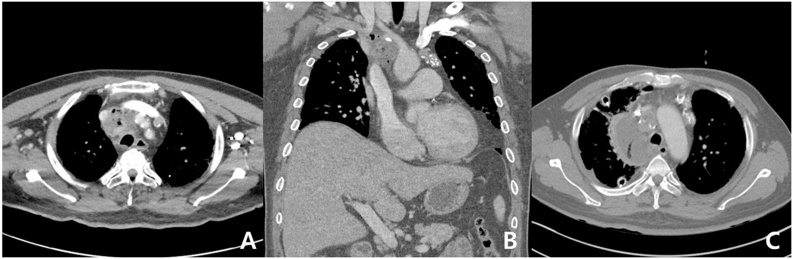
(A, B) Chest CT, performed 21 days after EBUS-TBNA, shows enlargement of the lymph node accompanied by the intranodal necrotic portion; (C) chest CT, performed 5 days after operation, shows loculated effusion with newly generated gas formation.

On chest CT scan, enlargement of the lymph node accompanied by the intranodal necrotic portion was found (Fig. 2A, B). The patient underwent surgery for debridement on the same day.

The operation was performed with VATS under general anesthesia. A layer accompanied by calcification was formed around the swollen lymph node, and a small amount of fluid was observed during the dissection. Cytology, culture, and biopsy were performed, and the 28-French chest tube was inserted, and the operation was completed. After the operation, the patient had stable vital signs, with mild fever, and broad-spectrum antibiotics were administered intravenously.

On the 5th postoperative day, the fever persisted and the chest x-ray showed an increase in shadow around the surgical site; therefore, the chest CT scan was re-evaluated. A loculated effusion with newly generated gas formation was observed, and empyema was suspected; therefore, the patient underwent a reoperation (Fig. 2C). In the previous lymph node enlargement site, a space with a large amount of pus was found without inflammatory findings. All fluid was removed, and massive irrigation was performed using saline. After the operation, the patient had no additional fever and blood test results also normalized. The pathological examination of the biopsy obtained during the first surgery revealed mature adipose tissue with focal fat necrosis, without other malignant or abnormal findings. The patient was discharged 8 days after the second surgery without any clinical signs of fever or inflammation.

## 3 Discussion

EBUS-TBNA was first introduced in the 2000s and was used for staging lung cancer, mainly to detect metastasis of mediastinal lymph nodes [4,5]. However, since then, it has been widely applied for the determination of the clinical stage of lung cancer, and diagnosis of hilar lymphadenopathy or mediastinal disease, such as sarcoidosis or tuberculous lymphadenopathy [6,7].

Before the introduction of EBUS-TBNA, mediastinoscopy was mainly used to diagnose these diseases; however, complications have always been a problem [8]. The advantage of EBUS-TBNA over surgery is the possibility to be performed under local anesthesia, relatively short operation time, short periods of hospital stay, and cosmetic effect. In addition, since it is performed by a minimally invasive method, it is convenient to implement and shows a relatively high diagnosis rate.

The most common complication, bleeding, usually resolves spontaneously, although cases that required intervention or have resulted in death due to major bleeding have been reported [9,10]. If the lesion is located near a major vessel or is anatomically difficult to access, the approach is possible through VATS or mediastinoscopy, although these methods should be performed under general anesthesia. In addition, various factors, such as patient's condition, discontinuation of antiplatelet drug before the procedure, or operator's skill level, can increase the risk of bleeding related to EBUS-TBNA [11,12].

Pneumothorax is also one of the complications that can occur after the procedure. Many cases naturally resolve after conservative management, although in some situations, surgical correction may be required due to continuous air leakage after tube thoracostomy or chest tube insertion [10]. In addition, in patients with conditions such as chronic obstructive pulmonary disease, severe complications such as tension pneumothorax may occur, requiring careful observation after the procedure and immediate treatment if necessary. Cases of airway obstruction caused by hematoma in the trachea related to the treatment site have been reported; therefore, close observation is very important.

Postoperative infections such as mediastinitis, pneumonia, mediastinal abscess, empyema, lung abscess, or sepsis have also been reported [[13], [14], [15], [16]]. Although of relatively low probability, mediastinitis that is not properly treated can lead to sepsis, requiring immediate diagnosis and treatment [17]. Therefore, when the nature of the lesion is suspected more as benign than malignant, for example, lesions with little enhancement of contrast on CT or accompanied by necrosis, another approach may be necessary.

Recently, a new tool for advanced radiological examination such as 3D analysis system (Fujifilm Synapse Vincent system, Fujifilm Corporation, Tokyo, Japan) has been actively used in clinical practice. If we can effectively predict the distinction between malignant and benign lesions through the active introduction of these methods, the possibility of such complications can be reduced.

In our case, when the lesion was confirmed by fever, increased inflammation, and CT scan, we immediately approached the patient and actively drained the lesion, and a good prognosis was obtained by using intravenous antibiotics after the surgery [18].

More case studies should be conducted to further analyze the efficacy of these treatments. In addition, in patients are at risk of infection, it is believed that active use of antibiotics before and after the procedure can be helpful, and the research and establishment of guidelines on this matter are necessary. The work has been reported in accordance with the SCARE criteria [19].

## 4 Conclusion

The importance of surgical procedures for mediastinitis caused by EBUS-TBNA was suggested. Further research and establishment of guidelines on this matter is necessary.

## Declaration of Competing Interest

The authors report no declarations of interest.

## Funding

None.

## Ethical approval

None.

## Consent

This research proceeded with all patient’s consents.

## Registration of research studies

None.

## Guarantor

Sung Joon Han, MD, PhD.

## Provenance and peer review

Not commissioned, externally peer-reviewed.

## CRediT authorship contribution statement

**Sung Joon Han:** Conceptualization, Methodology, Data curation, Writing - original draft, Visualization, Investigation. **Yooyoung Chong:** Supervision. **Man-shik Shim:** Supervision. **Woosik Han:** Supervision. **Hyun Jin Cho:** Supervision. **Shin Kwang Kang:** Supervision. **Jae Hyeon Yu:** Supervision, Writing - review & editing.
